# Management of infertility among primary health care physicians in Morocco: a cross-sectional study

**DOI:** 10.11604/pamj.2025.50.12.45189

**Published:** 2025-01-07

**Authors:** Sana El Adlani, Abdelhafid Benksim, Mohamed Cherkaoui

**Affiliations:** 1 Pharmacology, Neurobiology, Anthropobiology and Environment, Faculty of Sciences Semlalia, Marrakesh, Morocco,; 2 High Institute of Nursing Professions and Health Techniques, Marrakesh, Morocco

**Keywords:** General physicians, infertility, management, primary health care

## Abstract

**Introduction:**

the management of infertility in primary health care services can be the first opportunity to address the inability to conceive. The objective of this investigation is to explore practices, obstacles, and recommendations of general physicians (GPs) towards infertility management in Moroccan primary health care services (PHCSs).

**Methods:**

a cross-sectional study with a total population sample was conducted (325 GPs accepted to participate in our study) between December 2022 and October 2023. They were working in PHCSs of Marrakesh-Safi region. The questions were about the characteristics of GPs, infertility management, and suggestions to improve their management approaches for infertility. Statistical analyses were performed using Statistical Package for the Social Sciences version 25.

**Results:**

the results showed that infertility management in PHCSs was influenced by gender (p= 0.016), age (p=0.003), post-graduated period (p < 0.001), and years of experience (p < 0,001). Forty-four point three percent (44.3%) of physicians believed that infertility management is not an activity for PHCSs. Seventy point two percent (70.2%) of them did not investigate among infertile couples. However, just 13.8% of them never gave advice about improving fertility. Ninety-five point four percent (95.4%) reported that they never participated in infertility treatment. Communication with specialists after referral (OR= 8,044; p= 0.050) and lack of knowledge about infertility (OR= 16,173; p<0.001) influenced infertility management by GPs.

**Conclusion:**

general physicians in our study had positive attitudes about infertility management in PHCSs. Results showed that there is a need to improve infertility management and to develop standard guidelines and continuing education programs about infertility is a pivotal way to promote infertility management in PHCSs.

## Introduction

The prevalence of infertility is on the increase worldwide. It has become one of priority of public health. It is a reproductive health disease, which can affect the relationship of a couple and their quality of life, especially in developing countries like Africa [1]. In general, specialized care from fertility experts is often necessary for cases that are more complex. However, the role of general practitioners in the initial evaluation and management of infertility is crucial [2]. Unfortunately, primary health care services (PHCSs) are insufficient in this area, especially in underdeveloped or developing countries [2]. General physicians (GPs) serve as the first point of contact for infertile couples. Their role encompasses various aspects, in the first instance; they conduct comprehensive evaluations, considering medical history, lifestyle factors, and basic diagnostic tests to identify potential causes of infertility, emotional support, and timely referrals to specialized care [3]. This initial assessment involves examining both partners to gauge their overall health and potential contributing factors [4]. Also, GPs can initiate basic treatments, such as prescribing ovulation-inducing medications, and hormonal therapies, or addressing minor hormonal imbalances, in cases where straightforward interventions may be beneficial [3-5].

Their role in addressing initial concerns and guiding patients through the early stages of infertility underscores the significance of a multidisciplinary approach in ensuring comprehensive care for individuals and couples facing fertility challenges [3-5]. The orientations of the World Health Organization in 1968 and in the Alma Ata Declaration of 1978 fixed the correlation of infertility services with primary health care [6,7]. Usually, general physicians believed that the issue of infertility did not have a place during routine examinations is a cause for concern [2]. In this sense, a few physicians viewed the evaluation of infertile cases as appropriate in primary health care. Moreover, they felt less confident in performing investigations about Infertility [2]. The reasons behind the difficult management of infertility in primary health care were a) lack of laboratory investigations in primary care, b) lack of devices in primary care, and c) lack of communication with specialists after referral. All those results corroborate with the same studies in Germany, England, Iran, and Egypt [2,8,9]. Furthermore, in sub-Saharan Africa, infertility services are primarily accessible through private providers. However, this reliance on private care comes with high costs, widening the gap in accessing fertility treatments for everyone [10].

It is why the first visit to PCHS presents an important chance to discuss fertility concerns [11]. Consistent support throughout fertility treatment is crucial, particularly for this sensitive group seeking understanding and assistance [11]. This involves maintaining open lines of communication between patients and healthcare professionals. The Moroccan Health Plan 2024-2030 was developed to promote sexual and reproductive health services for all, including management of infertility in PHCSs and assisted reproductive technology (ART) [12]. However, despite the endeavors of infertility care advocates and the implementation of a law regulating infertility services in 2019, infertility services are still in a nascent stage of development, hindered by various obstacles [12]. Yet, few investigations have explicitly treated infertility in Morocco, especially the management of infertility in PHCSs. The objective of this study is to explore attitudes, practices, and obstacles related to this approach and to explore the management of infertility among PHCSs in Morocco. The principal question of our research was: what barriers do GPs of PHCSs face in managing infertility, including access to resources, lack of specialized training, time constraints, and their attitudes?

## Methods

**Study design and sitting:** a cross-sectional study was carried out between December 2022 and October 2023, in Marrakesh-Safi region in Morocco. Two thousand two hundred and sixty-six (2266) GPs were working in primary care public services of this region [13]. It is crucial to mentioned that in this investigation, we did not take “area of practice” as an independent variable, because only 5% of GPs who are affected in rural public center, therefore there will be no significant association between area of practice and management of infertility. The study dates were established in collaboration with responsible in the relevant public services.

**Study population:** we used G-power as tool of calculating a sample size, with a power of 80%, an effect size β = 0.2; a significance level α = 0.05 and Y= total of GPs in the region 2 266. G-power is recommended for sample size and power calculations for various statistical methods. It determines the number of participants needed to obtain statistically significant result. G-Power helps to avoid under-sampling or over-sampling. Our sample was composed of 341 GPs, whereas, 16 physicians refused to participate to this study. So, the sample capacity to constitute the study was estimated at 325 doctors (95% of all GPs). We included all GPs who were working in PHCSs, expected for resident physicians and GPs temporarily assigned to the region. The recruitment of GPs was conducted either directly at the medical consultation office or through an electronic questioner. Data collection was carried after obtaining authorization from the health department of Marrakesh-Safi region. All GPs in PHCSs of Marrakech-Safi region were survey. However, medical student as well as GPs assigned temporarily to PHCSs in the region were excluded from this study.

**Materials and data collection:** physicians filled out questionnaires while a member of researcher was present, in order to clarify any questions that doctors found difficult to understand. Before the interview, we explained to participant the protocol of the study and obtained their consents. The questionnaire was the most appropriate data collection method to this study. The form included a semi-structured questionnaire with open and close questions in French languages, about characteristics of GPs and questions about management of infertility in PHCSs. In addition, the questionnaire included open questions about the suggestions to improve their managing approaches for infertility.

**Statistical analysis:** we conducted a statistical quantitative descriptive analysis for the questionnaire. Initially, we conducted content analysis for open questions. Subsequently, statistical analyses were executed using the Statistical Package for Social Sciences (version 25). Descriptive statistics were computed for all continuous variables. To explore relationships between categorical variables, we employed the Chi-square test. Associations were quantified using odds ratios (OR) with 95% confidence intervals (CI). The significance of individual coefficients was tested using Wald χ^2^ and β statistics to confirm the true values. In all analyses, differences were deemed significant when P<0,05.

**Ethics committee approval:** this study was approved by the Ethics committee. However, the committee's reference number is not applicable. We had also the authorization from Health authority.

## Results

The rate of female and male doctors of PHCSs were 57.5% and 42.5%, respectively. The average age was 42 years, while the average year of experience was 15 years. Our finding in Table 1 revealed several associations between the characteristics of general doctors and their behavior toward infertility management. Female doctors showed higher interest in infertility management compared to male doctors (p= 0.016). The physicians who graduated between (1999, 2009) were the category who evaluated infertility in PHCS (p=0.003). The results demonstrated that doctors aged between (49, and 58) years had a proactive attitude toward infertility management (p < 0.001). Moreover, the dependent variable was influenced by years of experience in PHCS (GPs with experiences between (11,20) years, p < 0.001). The result of multivariate analysis for the management of infertility by GPs in PHCSs is presented in Table 2. When participants were asked about their advice concerning fertility, just 13,8% declared had never given any type of advice about this topic. Whereas, the advice about fertility significantly influenced infertility management in PHCSs (lifestyle advice: OR = 3,671; p= 0.095, importance of laboratory analysis: OR = 107,500; p < 0.001, assisted reproductive technologies: OR = 15,114; p < 0.001). About laboratory and radiological investigation, only hormonal assessment was significantly associated with infertility management in PHCSs (OR= 16,985; p < 0.001), while spermogram and hysterosalpingography did not show significance (OR = 16,985; p < 0.001) and (OR =1,224; p=0.807), respectively.

**Table 1:** characteristics of general physicians according to behavior toward management of infertility

Characteristics		Frequency N=325 (%)	Practice of infertility management		Kh2	P-value
			P n (%)	NP n’(%)		
**Gender**	Female	187 (57,5)	46(47,4)	141(61,8)	5,791	0.016
	Male	138 (42,5)	51(52,6)	87(38,2)		
**Age groups**	(29, 38)	47 (14.5)	04 (4.1)	43 (18.9)	21,804	0.000
	(39, 48)	69 (21.2)	13(13.4)	56 (24.6)		
	(49, 58)	139 (42.8)	51 (52.6)	88 (38.6)		
	Sup 58	70 (21.5)	29 (29.9)	41(18)		
**Post graduation period (year)**	>1988	20 (6.2)	08(8.2)	12 (5.3)	13,650	0.003
	(1988, 1998)	69 (21.2)	21 (21.6)	48 (21.1)		
	(1999, 2009)	127 (39.1)	49 (50.5)	78 (34.2)		
	(2010, 2020)	109 (33.5)	19 (19.6)	90(39.5)		
**Years of experience in PCHSs**	(1, 10)	130 (40)	18(18,6)	112(49,1)	30,404	0.000
	[11, 20]	103 (31.7)	48 (49.5)	55 (24.1)		
	(21, 30)	67 (20.6)	23 (23.7)	44 (19.3)		
	(31, 40)	25 (7.7)	08 (8.2)	17 (7.1)		

P: practice; NP: no practice; Kh2: Chi square test; Statistical significance at<0,005;PCHSs: primary care health services

**Table 2:** multivariate analysis results for management of infertility by general physicians in primary care health services

Variables		β	OR (CI 95%)	P-value
**Advice for couples**	Life>	1,300	3,671 (0,797; 16,899)	0.095
	ART	2,716	15,114 (3,46; 64,426)	0.000
	Laboratory analysis	4,677	107, 500 (13,467; 858,112)	0.000
**Laboratory and radiological investigation**	Hormonal assessment	2,832	16,985(7,500; 38,467)	0.000
	Spermogram	-19,902	NS	NS
	Hysterosalpingography	0,202	1,224 (0,241; 6,226)	0.807
**Referral to specialists**	Gynecologist	-1,464	0,232 (0.123; 0.433)	0.000
	ART Center	1,236	3,441 (1,205; 9,822)	0.021
	Endocrinologist	-0,178	0,837 (0.417; 1,678)	0.616
**Etiologies of infertility cases managed**		-19,006	NS	NS
**Traitement of Infertility**	Traitement of STI/hormone/ ovulation inductors	0,504	1,665 (0.456; 6,007)	0.443
	Collaborate with gynecologist	2,296	9,933 (1,095; 90,098)	0.410

OR: odds ratio, CI: confidence intervals, statistical significance at p<0, 05, NS: not significant ART: assisted reproductive technologies

According to this data, referring to a gynecologist or to an endocrinologist had no significant association with the management of infertility. On the other hand, referring to ART public center (OR=3,441; p=0.021) and proceeding with infertility treatment in coordination with gynecologist management (OR=9,933; p=0.410) had a strong association with infertility. According to the information provided, various variables concerning the difficulties of GPs in managing infertility in PHCSs were reported in Table 3. Sixty-five point two (65.2%) of GPs affirmed that communication with specialists was not favorable to managing infertility. In this sense, the absence of feedback from specialists after referral influenced the management of fertility by GPs (OR= 8,044; p=0.050). In addition, lack of knowledge about infertility had a strong significant association with the management of infertility (OR= 16,173; p<0,001). The others difficulties declared by GPs (Unavailable official liaison form; list of specialists from both private and public sectors; unavailable information about planning of consultation in ART center) had no significant association. Table 4 shows proposals forwarded by GPs for the provision of better service to infertile cases in PHCSs. All proposals reveal significant associations with the improvement of infertility management in PCHS including developing a liaison form (OR= 17,571; p=0.001) and a management guide (OR= 8,613; p<0.001), along with sensitizing the population (OR=13,911; p<0.001), demonstrate particularly strong associations. Additionally, implementing a continuing education program also contributes positively to better service provision (OR=1,952; p=0.041).

**Table 3:** multivariate analysis results for difficulties of general physicians to manage infertilities in primary care health services

Variables	β	OR (CI 95%)	P-value
Communication with specialists not favorable	0.220	1,247 (0.252; 6,166)	0.787
Unavailable official reference sheet	0.918	2,504 (0.441; 14,209)	0.300
Unavailable list of specialists from both private and public sectors	-0.091	0.913 (0.104; 8,002)	0.934
No feedback from specialists after referral	2.085	8,044 (0.990; 65,416)	0.050
Unavailable information about planning of consultation in ART Center (Public hospital)	-0.214	0.808 (0.160; 4,076)	0.796
Level of knowledge in this field is insufficient	2.783	16,173 (8,619; 30,348)	0.000

OR: Odds ratio, CI: Confidence intervals, Statistical significance at p<0, 05, ART: Assisted reproductive technologies

**Table 4:** proposals forwarded by general physicians for the provision of better service to infertile cases in primary care health services

Proposals	Practice of infertility management		β	OR (CI 95%)	P-value
	P n (%)	NP n’(%)			
Developing a reference sheet for infertility cases	06 (6.2)	02 (0.9)	2,866	17,571 (3,321; 92,958)	0.001
Developing a guide of infertility management for PCHSs	25 (25.8)	17 (7.5)	2,153	8,613 (3,986; 18,614)	0.000
Continuing education program about infertility management	26 (26.8)	78 (34.2)	0,669	1,952 (1,028; 3,707)	0.041
Sensitizing/Informing the population about infertility management in PCHSs	19 (19.6)	08 (3.5)	2,633	13,911 (5,397;35,856)	0.000

OR: odds ratio, CI: confidence intervals, Statistical significance at p<0, 05; P: practice; NP: no practice; PCHSs: primary care health services

## Discussion

This study is the first epidemiological investigation among GPs, conducted in Morocco to explore infertility management in PHCSs, to identify the attitude, practice, and difficulties of GPs related to fertility treatment and their recommendation to improve the quality and effectiveness of infertility care for these health care services. Firstly, qualitative research is required in our investigation in order to understand in-depth the knowledge, attitude, and perception of GPs which can influence their practice in infertility management. Also, concerning inferring causality for the association reported in this article, we reported adjusted and unadjusted ORs to control for the potential effect of confounders. Finally, the sample size can be a bias in our study, so the size is important in similar studies and we take into consideration the elaborated description of the participants´ characteristics. In our study, the management of infertility in PCHSs was influenced by gender, graduation period, and years of experience as GPs. Results from a review of the literature showed that female physicians had usually positive attitudes about infertility management in PCHSs [14,15]. In the other hand, a study in Southwest Virginia demonstrated that doctors with an experience more than 20 years were more motivated to evaluate infertility in PCHSs [14]. Concerning the practice of infertility management, 44.3% of GPs in our study affirmed that management of infertility needs specific skills, so they believed that it is not an activity for PHCSs.

These results corroborate with various studies in developed or developing countries [14-16]. In parallel with this finding, a few doctors (13.8%) never gave advice about fertility or reproductive health like lifestyle, ART, and the importance of laboratory analysis. The more frequently that general doctors assisted couples commonly involved counseling, providing advice, and education to promote fertility [3]. Seventy point two (70.2%) of GPs in this study didn´t investigate among infertile couples. However, GPs who investigated infertile couples had prescribed analysis like hormonal assessment, spermogram, and hysterosalpingography or they referred to gynecologists, endocrinologists, and ART public centers. In addition, a great proportion of GPs (95,4%) reported that they never participated in infertility treatment, on the other hand minority had prescribed treatment for sexually transmitted infection (STI), hormone therapy, and ovulation inductors. The most stated reason given, about their attitude and practice, was a lack related to infertility management and dissatisfaction about logistic support concerning referral and communication with specialists.

In worldwide, health strategies make available guidelines to define the appropriate investigation and management of infertility [17]. In Morocco, for the same reason; government, civil society, and the United Nations Population Fund mobilize all activities to promote infertility management as a public health [18]. Unfortunately, in PHCSs, there is no clinical guidelines or programs about infertility management [2,15-18]. Other barriers encountered by doctors in PHCSs during infertility management arose from reasons not directly connected to the patients including lack of knowledge, inadequacy in the supply of logistics and lack of communication [16-18]. In a similar way to the reports in various studies [2,19,20], to empower this care in PHCSs, GPs of this emphasis proposed: developing a liaison form, and infertility management guide, implementing a continuing education program about infertility, and sensitizing the population that infertility management can be provided in PCHSs. In this sense, supporting primary health care services and providing evidence-based training regarding infertility management are required to improve the attitude and practice of GPs towards infertility management.

## Conclusion

The finding of our current study can be used by the Ministry of Health to improve infertility management in PHCSs by GPs. This conclusion is from practices, difficulties and recommendations reported by GPs who participated to our investigation. It is crucial to have sufficient knowledge and positive attitude to practice the management of infertility in PHCSs. In this sense, when infertility is being adequately managed in PHCSs before referral to specialist or ART center, the quality of services and treatment among infertile couple will be better, efficient and effective. So, our perspective is to develop a standard guideline and continuing education program concerning infertility management in PHCSs.

### What is known about this topic

- The management of infertility should be practiced by general physicians in primary health care services before referral to specialists.

### What this study adds

- Usually, Moroccan general physicians in primary health care services did not investigate among infertile couples; this activity faced many obstacles like: attitudes of general physicians, lack of knowledge, deficiency in communication tools with specialists and insufficiency standard guideline.
